# Stronger associations of abdominal obesity-related triglyceride-glucose indices with type 2 diabetes compared to general obesity-related indices in community-dwelling Chinese adults: a cross-sectional study

**DOI:** 10.3389/fendo.2025.1526849

**Published:** 2025-06-10

**Authors:** Xiaoting Lu, Shanshan Chen, Cheng Wang, Diaozhu Lin, Fengyi He, Xiuhong Lin, Hongshi Wu, Ping Liang, Li Yan, Meng Ren, Chaogang Chen

**Affiliations:** ^1^ Department of Clinical Nutrition, Sun Yat-sen Memorial Hospital, Sun Yat-sen University, Guangzhou, China; ^2^ Department of Endocrinology, Sun Yat-sen Memorial Hospital, Sun Yat-sen University, Guangzhou, China

**Keywords:** triglyceride-glucose index, obesity-related TyG indices, type 2 diabetes, abdominal obesity, middle-aged and older Chinese adults, cross-sectional study

## Abstract

**Objectives:**

The relationships between the triglyceride-glucose (TyG) index, TyG-related parameters of different obesity phenotypes and the risk of type 2 diabetes (T2D) remain unclear. We aimed to determine associations between TyG index, obesity-related TyG parameters and T2D risk in Chinese adults.

**Methods:**

This cross-sectional study included 9489 participants aged ≥40 years from a large scale, community-based cohort study. Multivariable logistic regression was performed to estimate odds ratios (ORs) and 95% confidence intervals (CIs). The receiver operating characteristic (ROC) curve was employed to test and compare the predictive power of obesity-related TyG parameters across different phenotypes for the risk of T2D.

**Results:**

A total of 2081 (21.9%) participants with T2D were identified. When comparing with participants in the bottom quartile of TyG index, a heightened risk of T2D was observed among the highest quartile group, with an adjusted OR of 5.89 (95% CI: 4.98–6.98). Comparable relationships were found between obesity-related TyG indices and T2D, including TyG-waist circumference (TyG-WC), TyG-waist-to-height ratio (TyG-WHtR), TyG-body mass index (TyG-BMI) and TyG-body fat percentage (TyG-BFP). Abdominal obesity-related TyG indices had the highest predictive capability for T2D, with the area under the curve (AUC) was 0.711 (0.697–0.724) for TyG-WHtR and 0.705 (0.691–0.719) for TyG-WC, which was superior to the general obesity-related TyG indices, with the AUC were 0.683 (0.669–0.698) and 0.631 (0.616–0.646) for TyG-BMI and TyG-BFP, respectively.

**Conclusions:**

Our findings demonstrate a positive associations between TyG index, obesity-related TyG indices and risk of T2D. Abdominal obesity-related TyG indices had a better predictive value to diabetes than general obesity-related TyG indices.

## 1 Introduction

Diabetes stands as a prominent contributor to blindness, kidney failure, lower limb amputation and cerebrovascular accidents (1). It is estimated that 537 million adults suffer from diabetes in 2021, which is predicted to reach 783 million by 2045 (2). Type 2 diabetes (T2D) represents more than 95% of all diabetes cases. Interfering factors such as lifestyle, obesity, metabolic disorders are known to be recognized as key determinants of risks of T2D (3). Despite this, exploration into complex metabolism-related biomarkers on the risk of T2D has been limited.

Insulin resistance is the critical pathophysiological basic in development of T2D. The triglyceride-glucose (TyG) index, derived from the product of triglyceride (TG) and fasting plasma glucose (FPG) concentrations, has emerged as a non-insulin-based marker of insulin resistance in several studies (4–6). Given the widespread availability of TG testing compared to insulin, the TyG index may be a more convenient and practical method to assess insulin resistance in clinical practice (7), in comparison with the traditional indicator of homeostatic model assessment of insulin resistance (HOMA-IR) calculated using insulin and FPG levels. Furthermore, TyG index exhibit superior sensitivity in detecting insulin resistance in Chinese T2D patients compared to HOMA-IR (8). Associations between TyG index and prediabetes and T2D risk have been identified in previous studies (5, 9, 10). However, the robustness of TyG index and its-related indices remains controversial in the current studies (11–14).

Obesity is another modifiable and major cause for T2D, which usually interacts with other risk factors to elevate the risk of chronic conditions. Obesity could be categorized into general and abdominal adiposity, commonly characterized by body mass index (BMI), body fat percentage (BFP), waist circumference (WC), or waist-to-height ratio (WHtR). The latest research has demonstrated that general and abdominal obesity could discriminate people with or without hypertension (15). Abdominal obesity may pose a more important risk for pancreatic cancer compared to general adiposity (16). All of these suggests the differential impacts of distinct phenotypes of obesity on diseases outcomes. The existing studies have explored the relationships between TyG-BMI, TyG-WC, TyG-WHtR and T2D (17–19), but few of them were engaged in the interaction of TyG index and BFP on T2D.

Therefore, the present study aimed to examine the relationship between TyG index, as well as obesity-related TyG indices, and the risk of T2D within a substantial sample size of middle-aged and elderly population in Chinese community.

## 2 Methods

### 2.1 Study population

A cross-sectional design was undertaken in a community in Guangzhou, China, utilizing a subgroup of participants from the Risk Evaluation of cAncers in Chinese diabeTic Individuals: A lONgitudinal (REACTION) study, which was established as a multicenter prospective cohort to assess diabetes and cancer in the Chinese population. Detailed protocols of this study have been reported previously (20, 21). In short, a total of 10,104 residents aged ≥ 40 years were recruited via examination notices or home visits from June to November 2011, among which 9,916 signed written informed consent to participate. Study protocol was approved by the Ethical Committee of Sun Yat-sen Memorial Hospital of Sun Yat-sen University and Ruijin Hospital Ethics Committee of Shanghai Jiao Tong University School of Medicine with an approval number of 14 on March 10, 2011, and adhered to the declaration of Helsinki and its later amendments.

**Figure 1:**
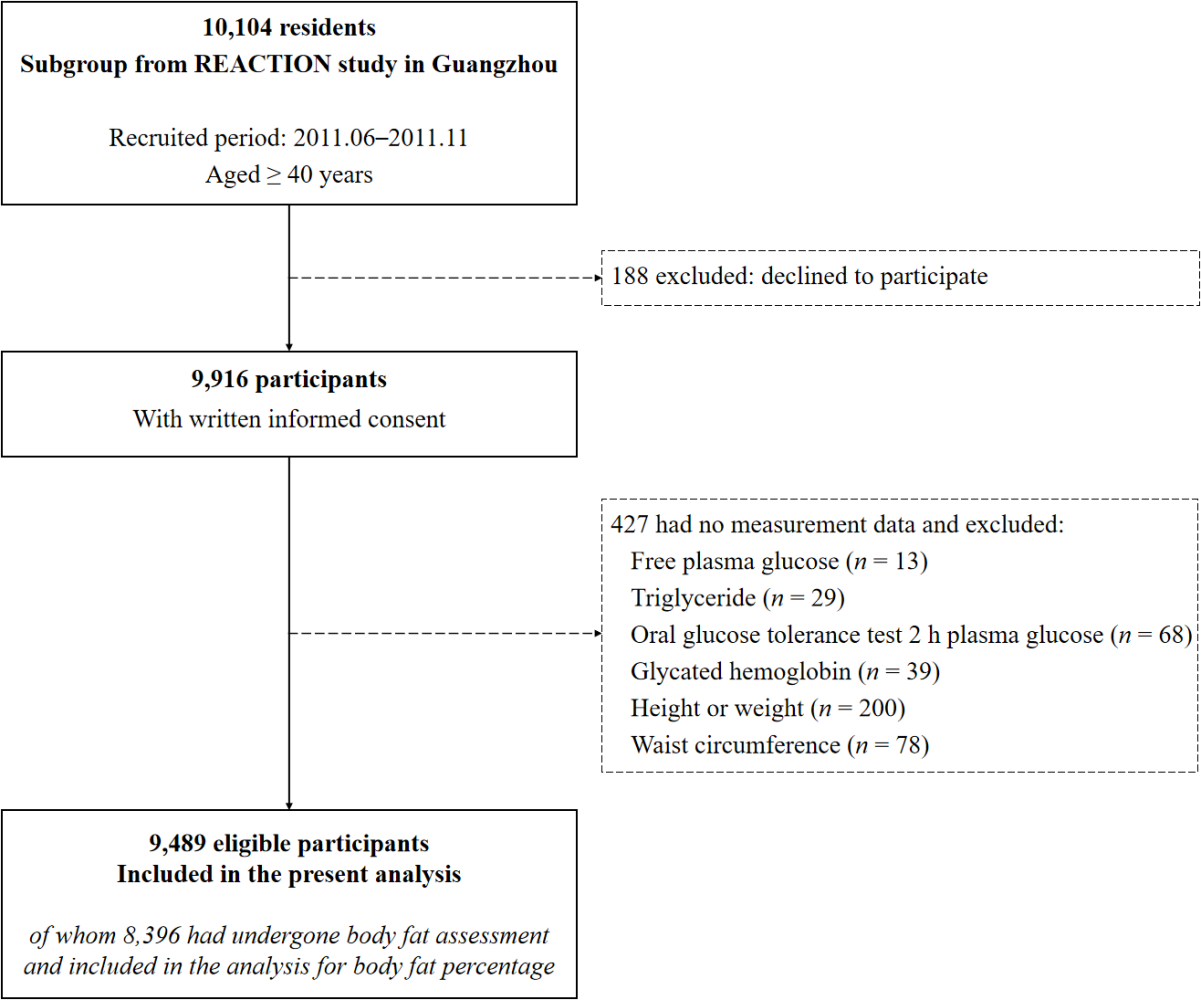
Flow diagram of the present study. REACTION study, the Risk Evaluation of cAncers in Chinese diabeTic Individuals: A lONgitudinal study.

For the current analysis, participants were further excluded if they had no measurement data of FPG (*n* = 13), TG (*n* = 29), oral glucose tolerance test 2 h plasma glucose (OGTT 2h-PG, *n* = 68), glycated hemoglobin (HbA1c, *n* = 39), height or weight, (*n* = 200), and WC (*n* = 78). Finally, 9,489 eligible individuals were included in the present analysis, with 8,396 having undergone body fat assessment and obtained BFP measurements (**Figure 1**).

### 2.2 Sociodemographic data collection and anthropometric assessments

A standardized questionnaire was conducted to gather sociodemographic information, lifestyle habits, and medication use. Education levels were categorized as less than high school, high school, and college or above, while occupation was stratified into light, medium and heavy physical labor. Smokers and alcohol drinkers were participants who smoked or drank regularly in the past six months.

Anthropometric measurements were conducted by experienced research personnel using standard procedure. BMI was calculated as weight/height squared (kg/m^2^), while WHtR was derived using the formula WC/height. Content of body fat was determined using a body fat meter (OMRON HBF-306, Omron Company, China) and BFP was calculated automatically. Blood pressure was recorded using the automated electronic device (OMRON, Omron Company, China). Individuals with elevated blood pressure (systolic ≥ 140 mmHg or diastolic ≥ 90 mmHg), self-reported diagnosis of hypertension by a clinician, or prescribed antihypertensive drugs were considered to existence of hypertension.

### 2.3 Blood sample collection and laboratory measurements

With a minimum of 10 hours of overnight fasting, peripheral venous blood was collected for laboratory analysis. FPG, fasting serum insulin (FINS), TG, total cholesterol (TC), high-density lipoprotein cholesterol (HDL-C), low-density lipoprotein cholesterol (LDL-C) were determined using the autoanalyzer (Beckman CX-7 Biochemical Autoanalyzer, Brea, CA, USA). HbA1c was measured by high-performance liquid chromatography (Bio-Rad, Hercules, CA). OGTT was carried out according to standard procedure and OGTT 2h-PG was determined. TyG index and obesity-related TyG indices were defined as described previously (22, 23): TyG = ln (TG [mg/dL] × FPG [mg/dL]/2), TyG-BMI = TyG × BMI, TyG-WC = TyG × WC, TyG-WHtR = TyG × WHtR, TyG-BFP = TyG × BFP.

### 2.4 Outcome definition

T2D was defined (24) if any of the following conditions was met: (1) FPG ≥7.0 mmol/L, (2) OGTT 2h-PG ≥11.1 mmol/L, (3) HbA1c ≥ 6.5%, (4) a self-reported diagnosis of diabetes by a clinician, (5)the use of diabetic medications, including insulin. Prediabetes was defined as the existence of one of the following: (1) concentrations of FPG were between 6.1 mmol/L and 7.0 mmol/L, (2) concentrations of OGTT 2h-PG were between 7.8 mmol/L and 11.1 mmol/L, (3) HbA1c was between 5.7% and 6.5%. Impaired glucose metabolism encompassed the presence of either T2D or prediabetes.

### 2.5 Statistical analysis

Participants were clustered into fours groups based on quartiles of TyG index. Continuous variables were described as mean and standard deviation (SD), whereas categorical variables were summarized by counts and frequencies, respectively. Comparisons of basic characteristics, including demographic, anthropometric, lifestyles, diseases information and laboratory measurements across TyG index quartiles, were conducted via one-way ANOVA, Kruskal-Wallis tests or Pearson’s Chi-Squared tests as appropriate.

Logistic regression was employed to investigate the relationship between both TyG index and obesity-related TyG indices (including TyG-BMI, TyG-WC, TyG-WHtR, and TyG-BFP), and T2D risk without adjustment in model 1. Model 2 adjusted for non-modifiable factors including sex (men, women) and age (continuous). Model 3 further adjusted for modifiable risk factors of T2D, including education levels (categorized as mentioned above), occupation (categorized as mentioned above), smoking (smoker, non-smoker), alcohol drinking (alcohol drinker, non-alcohol drinker) and BMI (continuous). Odds ratios (ORs) and 95% confidence intervals (CIs) were calculated, with the lowest quartile serving as the reference. Linear trends were assessed by replacing quartiles of TyG index with median values within subgroups and assigning as continuous variables in the models. Linear regression was also conducted to assess relationships between TyG index, obesity-related TyG-parameters, and indicators of glucose metabolism without and with adjustment for potential covariates mentioned above. Stratified analysis was performed to explore whether the correction between TyG index quartiles and T2D risk varied among subjects with different characteristics, with interactions estimated by including multiplicative interaction terms. The receiver operating characteristic (ROC) curve was performed to assess the predictive capability of TyG index and obesity-related TyG indices for T2D risk. Pearson’s Correlation analysis was conducted to explore relationships among TyG index and obesity-related TyG indices. All of the correlation coefficients calculated were greater than 0.4, indicating moderate to strong positive correlations among them (**Supplementary Table S1**). Missing covariates data were handled through the multiple imputation method.

All statistical analyses were performed using R 4.4.2 and were two-sided. *P* < 0.05 was considered statistically significant.

## 3 Results

### 3.1 Basic characteristics

Of the 9,489 study participants, there were 2,692 (28.4%) men and 6,797 (71.6%) women. Mean age was 56.0 ± 8.0 years. **Table 1** listed the basic characteristics of participants stratified by quartiles of TyG index. Participants in the highest quartile were older, had higher BMI, BFP, WC and blood pressure, high levels of FPG, OGTT 2h-PG, HbA1c, FINS, TC, TG and LDL-C, had lower education levels and lower HDL-C levels (all *P* < 0.001, all *P*-trend < 0.001) compared to those in the lowest quartile. Participants in the higher quartiles of TyG index tended to be men, smokers, and had high prevalence of T2D, impaired glucose metabolism and hypertension (all *P* < 0.001, all *P*-trend < 0.001). No significant differences were noted in alcohol drinking status among participants with various quartiles of TyG index (*P* = 0.937, *P*-trend = 0.924).

**Table 1:** Basic characteristics of participants by quartiles of TyG index^1^.

Characteristics	Total	Quartiles of TyG index				*P*	P-trend
		Q1 (n = 2505)	Q2 (n = 2501)	Q3 (n = 2498)	Q4 (n = 2501)		
Range of TyG index	6.16–12.07	≤8.70	8.27–8.63	8.64–9.04	≥9.05	<0.001	<0.001
Mean of TyG index	8.69 ± 0.61	7.99 ± 0.24	8.45 ± 0.10	8.82 ± 0.12	9.50 ± 0.43		
Age, years	56.0 ± 8.0	54.0 ± 7.7	55.7 ± 7.7	56.7 ± 7.9	57.5 ± 8.0	<0.001	<0.001
Sex, n (%)						<0.001	<0.001
Men	2692 (28.4)	563 (23.5)	615 (25.9)	681 (28.9)	833 (35.4)		
Women	6797 (71.6)	1835 (76.5)	1763 (74.1)	1677 (71.1)	1522 (64.6)		
Occupation, n (%)						<0.001	<0.001
Light labor	6999 (73.8)	1672 (69.7)	1751 (73.6)	1784 (75.7)	1792 (76.1)		
Medium labor	1426 (15.0)	444 (18.5)	354 (14.9)	321 (13.6)	307 (13.0)		
Heavy labor	1064 (11.2)	282 (11.8)	273 (11.5)	253 (10.7)	256 (10.9)		
Education levels, n (%)						<0.001	<0.001
Less than high school	3715 (39.2)	845 (35.2)	897 (37.7)	944 (40.0)	1029 (43.7)		
High school	4893 (51.6)	1319 (55.0)	1266 (53.2)	1192 (50.6)	1116 (47.4)		
College or above	881 (9.3)	234 (9.8)	215 (9.0)	222 (9.4)	210 (8.9)		
Smoking status, n (%)						<0.001	<0.001
Non-smoker	7750 (81.7)	2038 (85.0)	1976 (83.1)	1925 (81.6)	1811 (76.9)		
Smoker	1739 (18.3)	360 (15.0)	402 (16.9)	433 (18.4)	544 (23.1)		
Alcohol drinking status, n (%)						0.937	0.924
Non-alcohol drinker	6832 (72.0)	1732 (72.2)	1700 (71.5)	1701 (72.1)	1699 (72.1)		
Alcohol drinker	2657 (28.0)	666 (27.8)	678 (28.5)	657 (27.9)	656 (27.9)		
Height, cm	158.3 ± 7.5	158.0 ± 7.3	158.1 ± 7.3	158.2 ± 7.6	159.0 ± 7.9	<0.001	<0.001
Weight, kg	59.3 ± 9.6	56.2 ± 8.9	57.9 ± 8.9	60.2 ± 9.6	62.9 ± 9.6	<0.001	<0.001
BMI, kg/m^2^	23.6 ± 3.1	22.5 ± 3.1	23.1 ± 3.0	24.0 ± 3.1	24.8 ± 2.9	<0.001	<0.001
WC, cm	81.6 ± 9.2	77.7 ± 8.7	80.1 ± 8.8	82.9 ± 8.8	85.8 ± 8.3	<0.001	<0.001
Hip circumference, cm	94.0 ± 7.0	92.4 ± 6.7	93.3 ± 6.9	94.5 ± 7.0	95.7 ± 7.0	<0.001	<0.001
WHtR	0.52 ± 0.06	0.49 ± 0.05	0.51 ± 0.06	0.52 ± 0.05	0.54 ± 0.05	<0.001	<0.001
Body fat percentage, %	29.4 ± 6.0	27.9 ± 6.1	29.0 ± 6.1	30.0 ± 5.8	30.5 ± 5.8	<0.001	<0.001
SBP, mmHg	126.1 ± 16.5	120.4 ± 15.4	124.2 ± 15.6	127.4 ± 16.1	132.5 ± 16.5	<0.001	<0.001
DBP, mmHg	75.3 ± 9.8	72.4 ± 9.6	74.3 ± 9.6	76.0 ± 9.4	78.6 ± 9.6	<0.001	<0.001
FPG, mmol/L	5.7 ± 1.3	5.1 ± 0.6	5.4 ± 0.7	5.7 ± 1.0	6.6 ± 2.1	<0.001	<0.001
OGTT 2h-PG, mmol/L	8.1 ± 3.1	6.9 ± 2.1	7.5 ± 2.3	8.3 ± 2.9	9.8 ± 3.9	<0.001	<0.001
HbA1c, %	6.0 ± 0.9	5.7 ± 0.5	5.9 ± 0.5	6.0 ± 0.7	6.5 ± 1.4	<0.001	<0.001
FINS, μU/ml	8.3 ± 5.8	6.2 ± 3.6	7.3 ± 5.9	8.8 ± 5.1	10.9 ± 7.1	<0.001	<0.001
TC, mmol/L	5.2 ± 1.3	4.5 ± 1.3	5.2 ± 1.1	5.5 ± 1.1	5.7 ± 1.2	<0.001	<0.001
TG, mmol/L	1.6 ± 1.2	0.7 ± 0.2	1.1 ± 0.2	1.5 ± 0.3	3.0 ± 1.7	<0.001	<0.001
HDL-C, mmol/L	1.3 ± 0.4	1.4 ± 0.4	1.4 ± 0.4	1.3 ± 0.3	1.2 ± 0.3	<0.001	<0.001
LDL-C, mmol/L	3.1 ± 1.0	2.7 ± 0.9	3.2 ± 0.9	3.4 ± 0.9	3.3 ± 1.0	<0.001	<0.001
TyG-WC	710.8 ± 107.6	621.2 ± 73.7	676.8 ± 75.5	731.4 ± 79.8	815.9 ± 90.1	<0.001	<0.001
TyG-WHtR	4.49 ± 0.67	3.94 ± 0.46	4.29 ± 0.48	4.63 ± 0.49	5.14 ± 0.56	<0.001	<0.001
TyG-BMI	205.6 ± 34.3	179.7 ± 25.4	195.4 ± 25.8	211.9 ± 27.5	235.9 ± 30.4	<0.001	<0.001
TyG-BFP	255.5 ± 57.8	223.2 ± 50.0	245.4 ± 51.4	264.7 ± 51.6	289.7 ± 56.0	<0.001	<0.001
Presence of hypertension, n (%)						<0.001	<0.001
No	7525 (79.8)	2096 (87.9)	1983 (83.7)	1842 (78.8)	1604 (68.5)		
Yes	1906 (20.2)	288 (12.1)	385 (16.3)	496 (21.2)	737 (31.5)		
Presence of type 2 diabetes, n (%)						<0.001	<0.001
No	7408 (78.1)	2187 (91.2)	2049 (86.2)	1834 (77.8)	1338 (56.8)		
Yes	2081 (21.9)	211 (8.8)	329 (13.8)	524 (22.2)	1017 (43.2)		
Presence of impaired glucose metabolism, n (%)						<0.001	<0.001
No	1877 (19.8)	813 (33.9)	528 (22.2)	356 (15.1)	180 (7.6)		
Yes	7612 (80.2)	1585 (66.1)	1850 (77.8)	2002 (84.9)	2175 (92.4)		
Status of glucose metabolism, n (%)						<0.001	<0.001
Normal	1877 (19.8)	813 (33.9)	528 (22.2)	356 (15.1)	180 (7.6)		
Prediabetes	5531 (58.3)	1374 (57.3)	1521 (64.0)	1478 (62.7)	1158 (49.2)		
Diabetes	2081 (21.9)	211 (8.8)	329 (13.8)	524 (22.2)	1017 (43.2)		

^1^ Data are mean ± SD or *n* (%).

TyG index, triglyceride-glucose index; Q1, first quartile; Q2, second quartile; Q3, third quartile; Q4, fourth quartile; BMI, body mass index; WC, Waist circumference; WHtR, waist-to-height ratio; SBP, systolic blood pressure; DBP, diastolic blood pressure; FPG, fasting plasma glucose; OGTT 2h-PG, oral glucose tolerance test 2 h plasma glucose; HbA1c, glycated hemoglobin; FINS, fasting serum insulin; TC, total cholesterol; TG, triglycerides; HDL-C, high-density lipoprotein cholesterol; LDL-C, low-density lipoprotein cholesterol; WC, waist circumference; BFP, body fat percentage.

### 3.2 TyG index, obesity-related TyG indices and risk of T2D

**Table 2** presented the relationships between TyG index quartiles, obesity-related TyG indices and T2D. As for TyG index, the number of cases of T2D were lowest in the bottom quartile and highest in the fourth quartile of TyG index. A positive correction between TyG index and T2D risk was identified with an unadjusted OR of 7.88 (95% CI: 6.69–9.27, *P*-trend < 0.001) in the highest quartile in comparison with the first quartile. Multivariable analyses, adjusted for sex and age, revealed an adjusted OR of 7.08 (95% CI: 6.00–8.35, *P*-trend < 0.001) in the highest TyG index quartile compared to the lowest. This association remained statistically significant after further adjusting for modifiable T2D risk factors, with a fully adjusted OR of 5.89 (95% CI: 4.98–6.98, *P*-trend < 0.001) in the fourth quartile. When considering TyG index as a continuous variable, the risk of T2D also sharply increased for each SD increment in TyG index, irrespective of potential covariate adjustments (fully adjusted OR = 2.07, 95% CI: 1.96–2.19, *P* < 0.001). With regard to obesity-related TyG indices, including TyG-BMI, TyG-WC, TyG-WHtR and TyG-BFP, similar findings were noted between obesity-related TyG indices and T2D without or with adjustments for potential covariates (all *P*-trend < 0.001). Consistent results were also found between TyG index, obesity-related TyG indices and risk of impaired glucose metabolism (**Supplementary Table S2**).

**Table 2:** Odds ratios and 95% CIs of T2D by quartiles of TyG and obesity-related TyG indices.

TyG index	Quartiles of TyG and obesity-related TyG indices				*P*-trend	Per SD increment	
	Q1	Q2	Q3	Q4			
TyG							
Case/total, *n*	211/2398	329/2378	524/2358	1017/2355			
Model 1^1^	1.00 (ref.)	1.66 (1.39, 2.00)	2.96 (2.49, 3.52)	7.88 (6.69, 9.27)	<0.001	2.24 (2.13, 2.37)	<0.001
Model 2^2^	1.00 (ref.)	1.55 (1.29, 1.87)	2.66 (2.23, 3.16)	7.08 (6.00, 8.35)	<0.001	2.21 (2.09, 2.33)	<0.001
Model 3^3^	1.00 (ref.)	1.47 (1.22, 1.77)	2.35 (1.97, 2.80)	5.89 (4.98, 6.98)	<0.001	2.07 (1.96, 2.19)	<0.001
Abdominal obesity-related TyG indices							
TyG-WC							
Case/total, *n*	210/2379	357/2369	563/2378	951/2363			
Model 1^1^	1.00 (ref.)	1.83 (1.53, 2.20)	3.20 (2.70, 3.80)	6.96 (5.91, 8.19)	<0.001	2.15 (2.04, 2.27)	<0.001
Model 2^2^	1.00 (ref.)	1.78 (1.48, 2.13)	2.98 (2.51, 3.54)	6.51 (5.51, 7.71)	<0.001	2.13 (2.01, 2.25)	<0.001
Model 3^3^	1.00 (ref.)	1.76 (1.45, 2.12)	2.93 (2.42, 3.54)	6.30 (5.11, 7.76)	<0.001	2.34 (2.17, 2.52)	<0.001
TyG-WHtR							
Case/total, *n*	214/2379	350/2367	529/2372	988/2371			
Model 1^1^	1.00 (ref.)	1.76 (1.47, 2.10)	2.90 (2.45, 3.44)	7.23 (6.14, 8.50)	<0.001	2.23 (2.11, 2.35)	<0.001
Model 2^2^	1.00 (ref.)	1.64 (1.37, 1.97)	2.65 (2.23, 3.15)	6.22 (5.28, 7.34)	<0.001	2.11 (2.00, 2.24)	<0.001
Model 3^3^	1.00 (ref.)	1.65 (1.37, 1.99)	2.67 (2.21, 3.23)	6.26 (5.09, 7.71)	<0.001	2.38 (2.20, 2.57)	<0.001
General obesity-related TyG indices							
TyG-BMI							
Case/total, *n*	237/2381	386/2373	538/2365	920/2370			
Model 1^1^	1.00 (ref.)	1.76 (1.48, 2.09)	2.66 (2.26, 3.14)	5.74 (4.90, 6.72)	<0.001	1.98 (1.88, 2.08)	<0.001
Model 2^2^	1.00 (ref.)	1.71 (1.44, 2.04)	2.58 (2.18, 3.05)	5.63 (4.80, 6.61)	<0.001	1.98 (1.88, 2.09)	<0.001
Model 3^4^	1.00 (ref.)	1.71 (1.44, 2.04)	2.58 (2.18, 3.05)	5.60 (4.77, 6.57)	<0.001	1.98 (1.87, 2.08)	<0.001
TyG-BFP							
Case/total, *n*	297/2100	364/2104	424/2103	721/2089			
Model 1^1^	1.00 (ref.)	1.27 (1.07, 1.50)	1.53 (1.30, 1.80)	3.20 (2.75, 3.73)	<0.001	1.65 (1.56, 1.75)	<0.001
Model 2^2^	1.00 (ref.)	1.48 (1.24, 1.76)	1.98 (1.66, 2.37)	4.28 (3.57, 5.13)	<0.001	1.86 (1.74, 1.99)	<0.001
Model 3^3^	1.00 (ref.)	1.30 (1.08, 1.55)	1.58 (1.30, 1.91)	2.86 (2.30, 3.56)	<0.001	1.67 (1.53, 1.83)	<0.001

^1^ Unadjusted. ^2^ Adjusted for age (continuous) and sex (men, women). ^3^ Adjusted additionally for education levels (less than high school, high school, college or above), occupation (light, medium, heavy physical labor), smoking status (smoker, non-smoker), alcohol drinking (alcohol drinker, non-alcohol drinker) and BMI (continuous). ^4^ Adjusted for covariates mentioned above except for BMI.

CI, confidence interval; T2D, type 2 diabetes; TyG index, triglyceride-glucose index; Q1, first quartile; Q2, second quartile; Q3, third quartile; Q4, fourth quartile; SD, standard deviation; WC, waist circumference; WHtR, waist-to-height ratio; BMI, body mass index; BFP, body fat percentage.

### 3.3 TyG index, obesity-related TyG indices and indicators of glucose metabolism

Linear corrections of TyG index, obesity-related TyG indices with glucose metabolism were shown in **Table 3**. Positive associations between TyG index and indicators of glucose metabolism, including FPG (*β* = 0.41, standard error (SE) = 0.01), OGTT 2h-PG (*β* = 0.85, SE = 0.03) and HbA1c (*β* = 0.23, SE = 0.01), were found (all *P* < 0.001), following adjustment for potential confounding factors. As for obesity-related TyG indices, identical positive associations were observed with FPG (*β*: 0.28–0.41, *P* < 0.001), OGTT 2h-PG (*β*: 0.50–0.91, *P* < 0.001) and HbA1c (*β*: 0.14–0.24, *P* < 0.001) after additionally adjusting for potential covariates.

**Table 3:** Associations between TyG index, obesity-related TyG indices and indicators of glucose metabolism.

TyG index	Model 1^1^			Model 2^2^			Model 3^3^		
	*β* (95% CI)	SE	*P*	*β* (95% CI)	SE	*P*	*β* (95% CI)	SE	*P*
FPG									
TyG	0.45 (0.42, 0.47)	0.01	<0.001	0.43 (0.41, 0.45)	0.01	<0.001	0.41 (0.39, 0.44)	0.01	<0.001
TyG-WC	0.36 (0.34, 0.39)	0.01	<0.001	0.35 (0.32, 0.37)	0.01	<0.001	0.41 (0.38, 0.44)	0.02	<0.001
TyG-WHtR	0.36 (0.33, 0.38)	0.01	<0.001	0.34 (0.32, 0.36)	0.01	<0.001	0.41 (0.38, 0.44)	0.02	<0.001
TyG-BMI	0.32 (0.29, 0.34)	0.01	<0.001	0.31 (0.28, 0.33)	0.01	<0.001	0.30 (0.28, 0.33)^4^	0.01	<0.001
TyG-BFP	0.21 (0.18, 0.23)	0.01	<0.001	0.29 (0.26, 0.32)	0.02	<0.001	0.28 (0.24, 0.32)	0.02	<0.001
OGTT 2h-PG									
TyG	0.96 (0.91, 1.01)	0.03	<0.001	0.91 (0.86, 0.96)	0.03	<0.001	0.85 (0.79, 0.90)	0.03	<0.001
TyG-WC	0.83 (0.77, 0.88)	0.03	<0.001	0.79 (0.74, 0.85)	0.03	<0.001	0.86 (0.79, 0.94)	0.04	<0.001
TyG-WHtR	0.87 (0.82, 0.93)	0.03	<0.001	0.81 (0.75, 0.86)	0.03	<0.001	0.91 (0.84, 0.98)	0.04	<0.001
TyG-BMI	0.76 (0.70, 0.81)	0.03	<0.001	0.73 (0.67, 0.78)	0.03	<0.001	0.72 (0.67, 0.77)^4^	0.03	<0.001
TyG-BFP	0.54 (0.48, 0.60)	0.03	<0.001	0.62 (0.55, 0.68)	0.03	<0.001	0.50 (0.42, 0.58)	0.04	<0.001
HbA1c									
TyG	0.26 (0.24, 0.27)	0.01	<0.001	0.24 (0.23, 0.26)	0.01	<0.001	0.23 (0.21, 0.24)	0.01	<0.001
TyG-WC	0.22 (0.21, 0.24)	0.01	<0.001	0.21 (0.20, 0.23)	0.01	<0.001	0.24 (0.21, 0.26)	0.01	<0.001
TyG-WHtR	0.23 (0.21, 0.24)	0.01	<0.001	0.21 (0.19, 0.23)	0.01	<0.001	0.24 (0.22, 0.26)	0.01	<0.001
TyG-BMI	0.20 (0.18, 0.21)	0.01	<0.001	0.19 (0.17, 0.21)	0.01	<0.001	0.19 (0.17, 0.20)^4^	0.01	<0.001
TyG-BFP	0.14 (0.12, 0.16)	0.01	<0.001	0.17 (0.15, 0.19)	0.01	<0.001	0.14 (0.11, 0.16)	0.01	<0.001

^1^ Unadjusted. ^2^ Adjusted for age (continuous) and sex (men, women). ^3^ Adjusted additionally for education levels (less than high school, high school, college or above), occupation (light, medium, heavy physical labor), smoking status (smoker, non-smoker), alcohol drinking (alcohol drinker, non-alcohol drinker) and BMI (continuous). ^4^ Adjusted for covariates mentioned above except for BMI.

TyG index, triglyceride-glucose index; Q1, first quartile; Q2, second quartile; Q3, third quartile; Q4, fourth quartile; CI, confidence interval; SE, standard error; FPG, fasting plasma glucose; WC, waist circumference; WHtR, waist-to-height ratio; BMI, body mass index; BFP, body fat percentage; OGTT 2h-PG, oral glucose tolerance test 2 h plasma glucose; HbA1c, glycated hemoglobin.

### 3.4 Interactions and stratified analyses

**Table 4** delineated the influences of TyG index on T2D risk across strata of selected potential risk factors. A statistically significant multiplicative interaction was demonstrated between quartiles of TyG index and sex on associations with T2D risk (*P*-interaction = 0.024). Stratified by sex, stronger associations of TyG index with T2D risk were found in women (OR = 6.51, 95% CI: 5.32–7.98, *P*-trend < 0.001) than in men (OR = 4.41, 95% CI: 3.23–6.01, *P*-trend < 0.001). No notable multiplicative interactions were identified between smoking status, alcohol drinking, different phenotypes of obesity and quartiles of TyG index (all *P*-interaction > 0.05). The influences of TyG index on risk of impaired glucose metabolism stratified by confounders mentioned above were shown in **Supplementary Table S3** and consistent results were found.

**Table 4:** Odds ratios and 95% CIs of T2D by quartiles of TyG index stratified by covariates^1^.

TyG index	*n*	Quartiles of TyG index				*P*-trend	*P*-interaction
		Q1	Q2	Q3	Q4		
Sex							0.024
Men	2692	1.00	1.46 (1.04, 2.07)	2.14 (1.55, 2.97)	4.41 (3.23, 6.01)	<0.001	
Women	6797	1.00	1.45 (1.16, 1.81)	2.38 (1.93, 2.93)	6.51 (5.32, 7.98)	<0.001	
Smoking status							0.058
Non-smoker	7750	1.00	1.42 (1.15, 1.74)	2.40 (1.97, 2.91)	6.27 (5.20, 7.57)	<0.001	
Smoker	1739	1.00	1.65 (1.08, 2.52)	1.99 (1.32, 3.01)	4.22 (2.86, 6.23)	<0.001	
Alcohol drinking status							0.683
Non-alcohol drinker	6832	1.00	1.39 (1.11, 1.73)	2.32 (1.89, 2.85)	5.86 (4.80, 7.14)	<0.001	
Alcohol drinker	2657	1.00	1.71 (1.21, 2.41)	2.38 (1.70, 3.33)	5.83 (4.21, 8.05)	<0.001	
WC^2^, cm							0.165
< 90 or 80	5044	1.00	1.44 (1.13, 1.84)	2.59 (2.04, 3.28)	5.66 (4.45, 7.19)	<0.001	
≥ 90 or 80	4445	1.00	1.57 (1.18, 2.09)	2.24 (1.72, 2.93)	6.27 (4.87, 8.09)	<0.001	
WHtR							0.170
≤ 0.5	3781	1.00	1.43 (1.07, 1.92)	2.93 (2.21, 3.89)	7.02 (5.24, 9.42)	<0.001	
> 0.5	5708	1.00	1.48 (1.16, 1.89)	2.07 (1.65, 2.60)	5.40 (4.35, 6.69)	<0.001	
BMI, kg/m^2^							0.599
< 24	5458	1.00	1.54 (1.22, 1.96)	2.69 (2.14, 3.38)	6.31 (5.05, 7.89)	<0.001	
≥ 24	4031	1.00	1.40 (1.04, 1.89)	2.19 (1.66, 2.89)	6.04 (4.65, 7.84)	<0.001	
BFP^3^, %							0.709
≤ 25 or 30	3469	1.00	1.54 (1.16, 2.07)	2.42 (1.81, 3.22)	6.10 (4.62, 8.07)	<0.001	
> 25 or 30	4927	1.00	1.46 (1.11, 1.92)	2.35 (1.82, 3.03)	6.26 (4.91, 7.99)	<0.001	

^1^ Adjusted for age (continuous) and sex (men, women), education levels (less than high school, high school, college or above), occupation (light, medium, heavy physical labor), smoking status (smoker, non-smoker), alcohol drinking (alcohol drinker, non-alcohol drinker) and BMI (continuous). Stratified factors were not included in the corresponding models. ^2^ Cut-off points of WC were 90 cm for men and 80 cm for women. ^3^ Cut-off points of BFP were 25% for men and 30% for women.

CI, confidence interval; T2D, type 2 diabetes; TyG index, triglyceride-glucose index; Q1, first quartile; Q2, second quartile; Q3, third quartile; Q4, fourth quartile; WC, waist circumference; WHtR, waist-to-height ratio; BMI, body mass index; BFP, body fat percentage.

### 3.5 The predictive value of TyG index, obesity-related TyG indices for T2D

**Figure 2:**
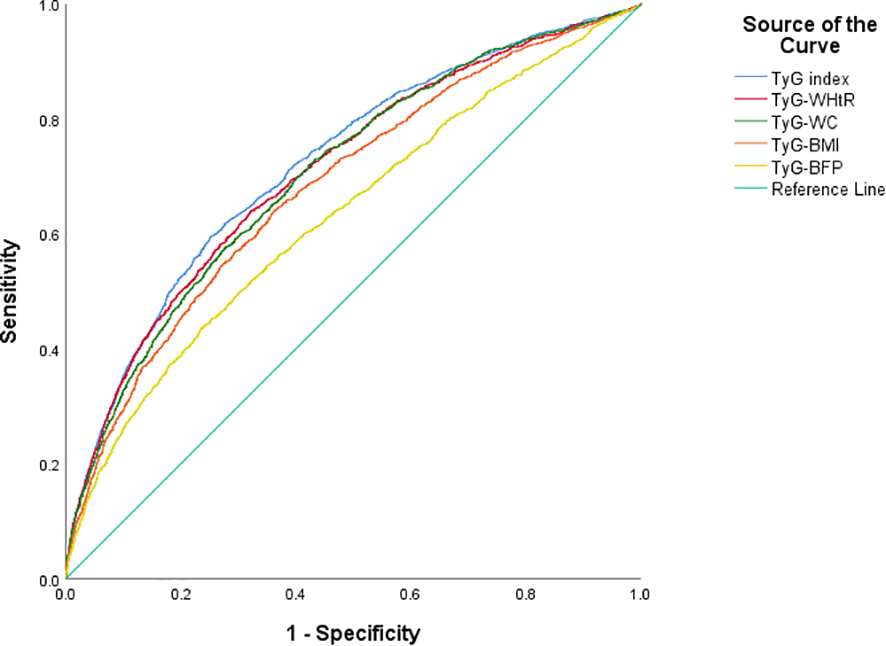
Predictive ability of T2D in TyG and obesity-related TyG indices of different phenotypes. T2D, type 2 diabetes; TyG index, triglyceride-glucose index; WHtR, waist-to-height ratio; WC, waist circumference; BMI, body mass index; BFP, body fat percentage.

ROC curve for TyG and obesity-related TyG indices were presented in **Figure 2**. TyG had the highest area under the curve (AUC = 0.722, 95% CI: 0.709–0.736), followed by TyG-WHtR (0.711, 95% CI: 0.697–0.724), TyG-WC (0.705, 95% CI: 0.691–0.719), TyG-BMI (0.683, 95% CI: 0.669–0.698) and TyG-BFP (0.631, 95% CI: 0.616–0.646). As for impaired glucose metabolism, similar results were observed in **Supplementary Figure S1**, with the AUC of 0.682 (95% CI: 0.669–0.696), 0.682 (95% CI: 0.668–0.695), 0.673 (95% CI: 0.659–0.686), 0.665 (95% CI: 0.651–0.678) and 0.614 (95% CI: 0.600–0.628) for TyG, TyG-WHtR, TyG-WC, TyG-BMI and TyG-BFP, respectively.

## 4 Discussion

In this representative cross-sectional study with 9,489 middle-aged and older Chinese adults, we delved into the corrections between T2D risk and both TyG index and obesity-related TyG indices. We observed that TyG index, as well as TyG-BMI, TyG-WC, TyG-WHtR and TyG-BFP, were positively associated with risk of T2D. These associations persisted even after accounting for potential confounders. Positive associations between TyG index, obesity-related TyG indices and FPG, OGTT 2h-PG and HbA1c were also found. In addition, TyG index had the higher predictive ability for T2D, followed by abdominal obesity-related TyG indices (TyG-WHtR and TyG-WC), and general obesity-related TyG indices (TyG-BMI and TyG-BFP).

A majority of previous researches have examined the associations between the TyG index and disorder glucose metabolism, including prediabetes and diabetes. In a cross-sectional study conducted in representative American adults, in comparison to the bottom group, participants with higher TyG index were associated with the higher risk of both prediabetes and diabetes (25). Another prospective cohort study undertaken in Japan also demonstrated a positive correction between TyG index and the risk of T2D (26). In the Chinese population, several studies conducted in geriatric (5, 27), rural area (9), or general individuals (28) found consistently that increasing TyG index was related with the higher incidence risk of prediabetes and diabetes. The above results revealed that TyG index served as a potential marker or predictor of risk of both prediabetes and T2D. Corresponding with these studies, the current investigation also observed a positive relationship between TyG index and T2D, as well as impaired glucose metabolism. Disorder of glucose and lipid metabolism was an crucial cause for diabetes. The increase of glucose and TG levels disrupted the function of pancreatic islet β cells through elevating reactive oxygen, inflammation, endoplasmic reticulum stress and ectopic fat deposition, therefore resulting to pancreatic dysfunction and insulin resistance (29–32). Therefore, the TyG index could be a complex and effective indicator to reflect diabetes or disorder of glucose metabolism, which aligned with the description of the present and previous researches mentioned above.

Obesity was another important and modifiable risk factor for diabetes. It was reported that obesity accounted for 52.2% of the disability-adjusted life-years (DALYs) linked to T2D in 2021, with a more than 20% increase since 1990 (33), which emphasized its significant impact on T2D. The existing studies have examined the relationships between T2D and obesity-related TyG indices, such as TyG-BMI, TyG-WC and TyG-WHtR (17–19). BMI was the most common indicator for measuring general obesity, and many studies combined it with the TyG index to investigate the correction between obesity-related TyG indices and impaired glucose metabolism. TyG-BMI was discovered to be associated with the risk of T2D, whatever prediabetes or general individuals from people of different ethnic groups (19, 34–37). Moreover, reduction of TyG-BMI via active management was possible to help to convert prediabetes into normoglycemia (38, 39). Body fat was additionally another indicator on behalf of general obesity, however, there was a paucity of studies that addressed the interaction of TyG index and body fat on T2D. In the present research, we found that the TyG-BFP index, the product of TyG and BFP, was as important as other effective obesity-indicator to predict T2D and impaired glucose metabolism. In regard to abdominal obesity, the latest research have found that general and abdominal adiposity could discriminate people with or without hypertension (15), which suggested that the abdominal adiposity, usually described by WC and WHtR, may be as a important risk factor as the general adiposity to diabetes. As the findings from previous studies shown, TyG-WC and TyG-WHtR were also effective markers to identify the diabetes risk (17, 18). Furthermore, they seem to be better indicators for predicting prediabetes in Indian (40) and Qatari (41) population in Asia. In this study, significant positive corrections were found between TyG-WC, TyG-WHtR, and risk of T2D and impaired glucose metabolism. TyG-WC and TyG-WHtR demonstrated superior predictive efficacy for diabetes than TyG-BMI and TyG-BFP, which were aligned with the previous findings.

Our study boasts notable strengths. It was undertaken in a representative, community-dwelling population with a wide range of age (equal or more than 40 years) in China. It was also a frontier research to combine different phenotypes of obesity, including general and abdominal obesity, with TyG index to discuss the relationship between obesity-related TyG indices and T2D, and compared their predictive value ulteriorly.

Several limitations need to be considered in our study. First, despite our efforts to adjust to potential risk factors, residual confounding cannot be eradicated fully. Moreover, the cross-sectional design constrained our ability to definitively rule out the possibility that reverse causality and residual confounding may have skewed our findings. Further prospective or interventional researches were required to solidify our understanding of associations between obesity-related TyG parameters and diabetes.

## 5 Conclusions

In conclusion, positive associations were observed between TyG index, as well as obesity-related TyG indices including TyG-BMI, TyG-WC, TyG-WHtR and TyG-BFP, and risk of T2D. Abdominal obesity-related TyG index had enhanced predictive value to diabetes than general obesity-related TyG index. Further large prospective researches were required to validate our findings.

## Data Availability

The data analyzed in this study is subject to the following licenses/restrictions: Data described in the article are available from the corresponding author upon reasonable request. Requests to access these datasets should be directed to Li Yan, yanli@mail.sysu.edu.cn.

## Glossary

T2D: type 2 diabetes
TyG index: triglyceride-glucose index
TG: triglyceride
FPG: fasting plasma glucose
HOMA-IR: homeostatic model assessment of insulin resistance
BMI: body mass index
BFP: body fat percentage
WC: waist circumference
WHtR: waist-to-height ratio
REACTION study: the Risk Evaluation of cAncers in Chinese diabeTic Individuals, A lONgitudinal study
OGTT 2h-PG: oral glucose tolerance test 2 h plasma glucose
HbA1c: glycated hemoglobin
FINS: fasting serum insulin
TC: total cholesterol
HDL-C: high-density lipoprotein cholesterol
LDL-C: low-density lipoprotein cholesterol
Q1: first quartile
Q2: second quartile
Q3: third quartile
Q4: fourth quartile
SD: standard deviation
OR: odds ratio
CI: confidence interval
ROC: receiver operating characteristic
SE: standard error
AUC: area under the curve
NHANES: National Health and Nutrition Examination Survey
NAGALA: NAfld in the Gifu Area, Longitudinal Analysis
DALYs: disability-adjusted life-years

## References

[1] World Health Organization. Diabetes . Available online at: https://www.who.int/news-room/fact-sheets/detail/diabetes (Accessed November 12, 2024).

[2] International Diabetes Federation. IDF Diabetes Atlas. 10th. Brussels, Belgium: International Diabetes Federation (2021). Available at: https://www.diabetesatlas.org/en/.

[3] Walford GA Ma Y Clish C Florez JC Wang TJ Gerszten RE. Metabolite profiles of diabetes incidence and intervention response in the diabetes prevention program. Diabetes. (2016) 65:1424–33. doi: 10.2337/db15-1063 PMC4839205 26861782

[4] Xuan X Hamaguchi M Cao Q Okamura T Hashimoto Y Obora A. U-shaped association between the triglyceride-glucose index and the risk of incident diabetes in people with normal glycemic level: A population-base longitudinal cohort study. Clin Nutr. (2021) 40:1555–61. doi: 10.1016/j.clnu.2021.02.037 33743291

[5] Wen J Wang A Liu G Wang M Zuo Y Li W. Elevated triglyceride-glucose (TyG) index predicts incidence of Prediabetes: a prospective cohort study in China. Lipids Health Dis. (2020) 19:226. doi: 10.1186/s12944-020-01401-9 33059672 PMC7565371

[6] Chen Z Wen J. Elevated triglyceride-glucose (TyG) index predicts impaired islet β-cell function: A hospital-based cross-sectional study. Front Endocrinol (Lausanne). (2022) 13:973655. doi: 10.3389/fendo.2022.973655 36246870 PMC9563389

[7] Massimino M Monea G Marinaro G Rubino M Mancuso E Mannino GC. The triglycerides and glucose (TyG) index is associated with 1-hour glucose levels during an OGTT. Int J Environ Res Public Health. (2022) 20:787. doi: 10.3390/ijerph20010787 36613109 PMC9819897

[8] Luo P Cao Y Li P Li W Song Z Fu Z. TyG index performs better than HOMA-IR in chinese type 2 diabetes mellitus with a BMI < 35 kg/m2: A hyperglycemic clamp validated study. Med (Kaunas). (2022) 58:876. doi: 10.3390/medicina58070876 PMC9325243 35888595

[9] Zhang M Wang B Liu Y Sun X Luo X Wang C. Cumulative increased risk of incident type 2 diabetes mellitus with increasing triglyceride glucose index in normal-weight people: The Rural Chinese Cohort Study. Cardiovasc Diabetol. (2017) 16:30. doi: 10.1186/s12933-017-0514-x 28249577 PMC5333419

[10] Wang Z Zhao L He S. Triglyceride-glucose index as predictor for future type 2 diabetes mellitus in a Chinese population in southwest China: a 15-year prospective study. Endocrine. (2021) 72:124–31. doi: 10.1007/s12020-020-02589-7 33433893

[11] Wang B Zhang M Liu Y Sun X Zhang L Wang C. Utility of three novel insulin resistance-related lipid indices for predicting type 2 diabetes mellitus among people with normal fasting glucose in rural China. J Diabetes. (2018) 10:641–52. doi: 10.1111/jdb.2018.10.issue-8 29322661

[12] Cybulska AM Schneider-Matyka D Wieder-Huszla S Panczyk M Jurczak A Grochans E. Diagnostic markers of insulin resistance to discriminate between prediabetes and diabetes in menopausal women. Eur Rev Med Pharmacol Sci. (2023) 27:2453–68. doi: 10.26355/eurrev_202303_31779 37013763

[13] Chamroonkiadtikun P Ananchaisarp T Wanichanon W. The triglyceride-glucose index, a predictor of type 2 diabetes development: A retrospective cohort study. Prim Care Diabetes. (2020) 14:161–7. doi: 10.1016/j.pcd.2019.08.004 31466834

[14] Darshan An V Rajput R Meena Mohini Garg R Saini S. Comparison of triglyceride glucose index and HbA1C as a marker of prediabetes - A preliminary study. Diabetes Metab Syndr. (2022) 16:102605. doi: 10.1016/j.dsx.2022.102605 36063676

[15] NCD Risk Factor Collaboration. General and abdominal adiposity and hypertension in eight world regions: a pooled analysis of 837 population-based studies with 7.5 million participants. Lancet. (2024) 404:851–63. doi: 10.1016/S0140-6736(24)01405-3 PMC7616775 39216975

[16] Maina JG Pascat V Zudina L Ulrich A Pupko I Bonnefond A. Abdominal obesity is a more important causal risk factor for pancreatic cancer than overall obesity. Eur J Hum Genet. (2023) 31:962–6. doi: 10.1038/s41431-023-01301-3 PMC10400602 37161092

[17] Zheng S Shi S Ren X Han T Li Y Chen Y. Triglyceride glucose-waist circumference, a novel and effective predictor of diabetes in first-degree relatives of type 2 diabetes patients: cross-sectional and prospective cohort study. J Transl Med. (2016) 14:260. doi: 10.1186/s12967-016-1020-8 27604550 PMC5015232

[18] Xuan W Liu D Zhong J Luo H Zhang X. Impacts of triglyceride glucose-waist to height ratio on diabetes incidence: A secondary analysis of A population-based longitudinal data. Front Endocrinol (Lausanne). (2022) 13:949831. doi: 10.3389/fendo.2022.949831 35937805 PMC9354460

[19] Wang X Liu J Cheng Z Zhong Y Chen X Song W. Triglyceride glucose-body mass index and the risk of diabetes: a general population-based cohort study. Lipids Health Dis. (2021) 20:99. doi: 10.1186/s12944-021-01532-7 34488806 PMC8420033

[20] Bi Y Lu J Wang W Mu Y Zhao J Liu C. Cohort profile: risk evaluation of cancers in Chinese diabetic individuals: a longitudinal (REACTION) study. J Diabetes. (2014) 6:147–57. doi: 10.1111/jdb.2014.6.issue-2 24237858

[21] Ning G. Risk Evaluation of cAncers in Chinese diabeTic Individuals: a lONgitudinal (REACTION) study. J Diabetes. (2012) 4:172–3. doi: 10.1111/j.1753-0407.2012.00182.x 22221801

[22] Li H Zuo Y Qian F Chen S Tian X Wang P. Triglyceride-glucose index variability and incident cardiovascular disease: a prospective cohort study. Cardiovasc Diabetol. (2022) 21:105. doi: 10.1186/s12933-022-01541-5 35689232 PMC9188105

[23] Song K Park G Lee HS Lee M Lee HI Choi HS. Comparison of the triglyceride glucose index and modified triglyceride glucose indices to predict nonalcoholic fatty liver disease in youths. J Pediatr. (2022) 242:79–85 e1. doi: 10.1016/j.jpeds.2021.11.042 34808224

[24] American Diabetes Association. Diagnosis and classification of diabetes mellitus. Diabetes Care. (2014) 37:S81–90. doi: 10.2337/dc14-S081 24357215

[25] Zhang L Zeng L. Non-linear association of triglyceride-glucose index with prevalence of prediabetes and diabetes: a cross-sectional study. Front Endocrinol (Lausanne). (2023) 14:1295641. doi: 10.3389/fendo.2023.1295641 38152130 PMC10751584

[26] Liu EQ Weng YP Zhou AM Zeng CL. Association between triglyceride-glucose index and type 2 diabetes mellitus in the Japanese population: A secondary analysis of a retrospective cohort study. BioMed Res Int. (2020) 2020:2947067. doi: 10.1155/2020/2947067 33490240 PMC7787715

[27] Fu X Liu H Liu J Li N Li L Ke D. Association between triglyceride-glucose index and the risk of type 2 diabetes mellitus in an older chinese population aged over 75 years. Front Public Health. (2021) 9:796663. doi: 10.3389/fpubh.2021.796663 35399348 PMC8989963

[28] He L Zheng W Li Z Kong W Zeng T. Association of four lipid-derived indicators with the risk of developing type 2 diabetes: a Chinese population-based cohort study. Lipids Health Dis. (2023) 22:24. doi: 10.1186/s12944-023-01790-7 36788551 PMC9930254

[29] Sondergaard E Nielsen S. VLDL triglyceride accumulation in skeletal muscle and adipose tissue in type 2 diabetes. Curr Opin Lipidol. (2018) 29:42–7. doi: 10.1097/MOL.0000000000000471 29135689

[30] Zhou M Zhu L Cui X Feng L Zhao X He S. The triglyceride to high-density lipoprotein cholesterol (TG/HDL-C) ratio as a predictor of insulin resistance but not of beta cell function in a Chinese population with different glucose tolerance status. Lipids Health Dis. (2016) 15:104. doi: 10.1186/s12944-016-0270-z 27267043 PMC4895977

[31] Li X Sun M Yang Y Yao N Yan S Wang L. Predictive effect of triglyceride glucose-related parameters, obesity indices, and lipid ratios for diabetes in a chinese population: A prospective cohort study. Front Endocrinol (Lausanne). (2022) 13:862919. doi: 10.3389/fendo.2022.862919 35432185 PMC9007200

[32] Lu X Xie Q Pan X Zhang R Zhang X Peng G. Type 2 diabetes mellitus in adults: pathogenesis, prevention and therapy. Signal Transduct Target Ther. (2024) 9:262. doi: 10.1038/s41392-024-01951-9 39353925 PMC11445387

[33] GBD 2021 Diabetes Collaborators. Global, regional, and national burden of diabetes from 1990 to 2021, with projections of prevalence to 2050: a systematic analysis for the Global Burden of Disease Study 2021. Lancet. (2023) 402:203–34. doi: 10.1016/S0140-6736(23)01301-6 PMC10364581 37356446

[34] Han Y Hu H Li Q Deng Z Liu D. Triglyceride glucose-body mass index and the risk of progression to diabetes from prediabetes: A 5-year cohort study in Chinese adults. Front Public Health. (2023) 11:1028461. doi: 10.3389/fpubh.2023.1028461 36817911 PMC9935616

[35] Navarro-González D Sánchez-Íñigo L Fernández-Montero A Pastrana-Delgado J Martinez JA. TyG index change is more determinant for forecasting type 2 diabetes onset than weight gain. Med (Baltimore). (2016) 95:e3646. doi: 10.1097/MD.0000000000003646 PMC4902528 27175686

[36] Low S Khoo KCJ Irwan B Sum CF Subramaniam T Lim SC. The role of triglyceride glucose index in development of Type 2 diabetes mellitus. Diabetes Res Clin Pract. (2018) 143:43–9. doi: 10.1016/j.diabres.2018.06.006 29936253

[37] Song B Zhao X Yao T Lu W Zhang H Liu T. Triglyceride glucose-body mass index and risk of incident type 2 diabetes mellitus in Japanese people with normal glycemic level: A population-based longitudinal cohort study. Front Endocrinol (Lausanne). (2022) 13:907973. doi: 10.3389/fendo.2022.907973 35909552 PMC9336540

[38] Yang H Kuang M Qiu J He S Yu C Sheng G. Relative importance of triglyceride glucose index combined with body mass index in predicting recovery from prediabetic state to normal fasting glucose: a cohort analysis based on a Chinese physical examination population. Lipids Health Dis. (2024) 23:71. doi: 10.1186/s12944-024-02060-w 38459527 PMC10921811

[39] Shao Y Hu H Cao C Han Y Wu C. Elevated triglyceride-glucose-body mass index associated with lower probability of future regression to normoglycemia in Chinese adults with prediabetes: a 5-year cohort study. Front Endocrinol (Lausanne). (2024) 15:1278239. doi: 10.3389/fendo.2024.1278239 38414822 PMC10898590

[40] Ramdas Nayak VK Nayak KR Vidyasagar S P R. Predictive performance of traditional and novel lipid combined anthropometric indices to identify prediabetes. Diabetes Metab Syndr. (2020) 14:1265–72. doi: 10.1016/j.dsx.2020.06.045 32688243

[41] Al Akl NS Haoudi EN Bensmail H Arredouani A. The triglyceride glucose-waist-to-height ratio outperforms obesity and other triglyceride-related parameters in detecting prediabetes in normal-weight Qatari adults: A cross-sectional study. Front Public Health. (2023) 11:1086771. doi: 10.3389/fpubh.2023.1086771 37089491 PMC10117653

