# Supplementary material for: Stronger associations of abdominal obesity-related triglyceride-glucose indices with type 2 diabetes compared to general obesity-related indices in community-dwelling Chinese adults: a cross-sectional study
Source: Front Endocrinol (Lausanne). 2025 Jun 10;16:1526849. doi: 10.3389/fendo.2025.1526849 (PMC12185517; doi:10.3389/fendo.2025.1526849)
Supplement: Supplementary file 1 [file DataSheet1.pdf]

Supplementary Material

Supplementary Table 1. Pearson's Correlation coefficients among TyG and obesity-related TyG indices.

r	TyG	TyG-WC	TyG-WHtR	TyG-BMI	TyG-BFP
TyG	1.000	0.714**	0.706**	0.642**	0.444**
TyG-WC		1.000	0.948**	0.850**	0.524**
TyG-WHtR			1.000	0.864**	0.645**
TyG-BMI				1.000	0.644**
TyG-BFP					1.000

**$P < 0.01$.

Abbreviations: TyG index, triglyceride-glucose index; WC, waist circumference; WHtR, waist-to-height ratio; BMI, body mass index; BFP, body fat percentage.

Supplementary Table 2. Odds ratios and 95% CIs of impaired glucose metabolism by TyG and obesity-related TyG indices.

TyG index	Quartiles of TyG and obesity-related TyG indices				*P* trend	Per SD increment	
	Q1	Q2	Q3	Q4			
TyG							
Case/total, *n*	1585/2398	1850/2378	2002/2358	2175/2355			
Model 1[1]	1.00 (ref.)	1.80 (1.58, 2.04)	2.88 (2.51, 3.32)	6.20 (5.21, 7.38)	<0.001	2.05 (1.93, 2.19)	<0.001
Model 2[2]	1.00 (ref.)	1.66 (1.46, 1.90)	2.56 (2.22, 2.96)	5.44 (4.55, 6.49)	<0.001	1.92 (1.80, 2.04)	<0.001
Model 3[3]	1.00 (ref.)	1.58 (1.38, 1.80)	2.25 (1.94, 2.60)	4.44 (3.70, 5.33)	<0.001	1.77 (1.66, 1.89)	<0.001
Abdominal obesity-related TyG indices							
TyG-WC							
Case/total, *n*	1612/2379	1819/2369	2028/2378	2153/2363			
Model 1[1]	1.00 (ref.)	1.57 (1.38, 1.79)	2.76 (2.39, 3.18)	4.88 (4.13, 5.76)	<0.001	1.91 (1.80, 2.03)	<0.001
Model 2[2]	1.00 (ref.)	1.51 (1.33, 1.73)	2.55 (2.20, 2.95)	4.56 (3.84, 5.42)	<0.001	1.85 (1.74, 1.97)	<0.001
Model 3[3]	1.00 (ref.)	1.39 (1.21, 1.60)	2.21 (1.86, 2.62)	3.65 (2.93, 4.54)	<0.001	1.81 (1.66, 1.97)	<0.001
TyG-WHtR							
Case/total, *n*	1600/2379	1819/2367	2004/2372	2189/2371			
Model 1[1]	1.00 (ref.)	1.62 (1.42, 1.84)	2.65 (2.30, 3.05)	5.86 (4.92, 6.97)	<0.001	2.00 (1.89, 2.13)	<0.001
Model 2[2]	1.00 (ref.)	1.49 (1.31, 1.70)	2.39 (2.07, 2.76)	4.88 (4.09, 5.83)	<0.001	1.87 (1.76, 1.99)	<0.001
Model 3[3]	1.00 (ref.)	1.40 (1.21, 1.61)	2.14 (1.80, 2.53)	4.08 (3.26, 5.11)	<0.001	1.88 (1.73, 2.05)	<0.001
General obesity-related TyG indices							
TyG-BMI							
Case/total, *n*	1623/2381	1813/2373	2012/2365	2164/2370			
Model 1[1]	1.00 (ref.)	1.51 (1.33, 1.72)	2.66 (2.31, 3.07)	4.91 (4.15, 5.80)	<0.001	1.88 (1.77, 1.99)	<0.001
Model 2[2]	1.00 (ref.)	1.46 (1.28, 1.67)	2.57 (2.22, 2.97)	4.79 (4.04, 5.68)	<0.001	1.86 (1.75, 1.97)	<0.001
Model 3[4]	1.00 (ref.)	1.46 (1.28, 1.67)	2.56 (2.21, 2.96)	4.78 (4.03, 5.66)	<0.001	1.86 (1.75, 1.97)	<0.001
TyG-BFP							
Case/total, *n*	1515/2100	1579/2104	1687/2103	1856/2089			
Model 1[1]	1.00 (ref.)	1.16 (1.01, 1.33)	1.57 (1.36, 1.81)	3.08 (2.60, 3.63)	<0.001	1.48 (1.40, 1.56)	<0.001
Model 2[2]	1.00 (ref.)	1.28 (1.10, 1.48)	1.79 (1.53, 2.10)	3.35 (2.79, 4.03)	<0.001	1.59 (1.49, 1.69)	<0.001
Model 3[3]	1.00 (ref.)	1.05 (0.90, 1.23)	1.27 (1.06, 1.52)	1.91 (1.51, 2.40)	<0.001	1.29 (1.18, 1.41)	<0.001

[1] Unadjusted. [2] Adjusted for age (continuous) and sex (men, women). [3] Adjusted additionally for education levels (less than high school, high school, college or above), occupation (light, medium, heavy physical labor), smoking status (smoker, non-smoker), alcohol drinking (alcohol drinker, non-alcohol drinker) and BMI (continuous). [4] Adjusted for covariates mentioned above except for BMI.

Abbreviations: CI, confidence interval; TyG index, triglyceride-glucose index; Q1, first quartile; Q2, second quartile; Q3, third quartile; Q4, fourth quartile; SD, standard deviation; WC, waist circumference; WHtR, waist-to-height ratio; BMI, body mass index; BFP, body fat percentage.

Supplementary Table 3. Odds ratios and 95% CIs of impaired glucose metabolism by TyG index stratified by covariates[1].

TyG index	*n*	Quartiles of TyG index				*P*-trend	*P*-interaction
		Q1	Q2	Q3	Q4		
Sex							0.032
Men	2692	1.00	1.52 (1.16, 2.00)	1.71 (1.30, 2.26)	3.26 (2.40, 4.44)	<0.001	
Women	6797	1.00	1.57 (1.35, 1.83)	2.47 (2.07, 2.94)	5.06 (4.01, 6.40)	<0.001	
Smoking status							0.076
Non-smoker	7750	1.00	1.51 (1.30, 1.74)	2.36 (2.00, 2.78)	4.62 (3.75, 5.69)	<0.001	
Smoker	1739	1.00	1.94 (1.37, 2.74)	1.82 (1.29, 2.57)	3.72 (2.53, 5.49)	<0.001	
Alcohol drinking status							0.405
Non-alcohol drinker	6832	1.00	1.60 (1.37, 1.87)	2.36 (1.99, 2.81)	4.90 (3.93, 6.10)	<0.001	
Alcohol drinker	2657	1.00	1.52 (1.18, 1.96)	1.96 (1.49, 2.59)	3.49 (2.51, 4.85)	<0.001	
WC[2], cm							0.524
< 90 or 80	5044	1.00	1.65 (1.40, 1.94)	2.25 (1.86, 2.72)	4.18 (3.25, 5.37)	<0.001	
≥ 90 or 80	4445	1.00	1.48 (1.18, 1.87)	2.26 (1.78, 2.86)	4.79 (3.64, 6.30)	<0.001	
WHtR							0.788
≤ 0.5	3781	1.00	1.56 (1.30, 1.86)	2.24 (1.81, 2.78)	3.99 (2.93, 5.44)	<0.001	
> 0.5	5708	1.00	1.61 (1.31, 1.96)	2.28 (1.86, 2.80)	4.72 (3.74, 5.96)	<0.001	
BMI, kg/m^2							0.314
< 24	5458	1.00	1.50 (1.28, 1.75)	2.29 (1.91, 2.74)	5.10 (3.98, 6.54)	<0.001	
≥ 24	4031	1.00	1.87 (1.46, 2.41)	2.54 (1.97, 3.27)	4.70 (3.56, 6.19)	<0.001	
BFP[3], %							0.405
≤ 25 or 30	3469	1.00	1.58 (1.30, 1.91)	2.12 (1.69, 2.65)	4.05 (3.02, 5.43)	<0.001	
> 25 or 30	4927	1.00	1.60 (1.31, 1.96)	2.49 (2.02, 3.08)	4.86 (3.79, 6.24)	<0.001	

[1] Adjusted for age (continuous) and sex (men, women), education levels (less than high school, high school, college or above), occupation (light, medium, heavy physical labor), smoking status (smoker, non-smoker), alcohol drinking (alcohol drinker, non-alcohol drinker) and BMI (continuous). Stratified factors were not included in the corresponding models. [2] Cut-off points of WC were 90 cm for men and 80 cm for women. [3] Cut-off points of BFP were 25% for men and 30% for women.

Abbreviations: CI, confidence interval; TyG index, triglyceride-glucose index; Q1, first quartile; Q2, second quartile; Q3, third quartile; Q4, fourth quartile; WC, waist circumference; WHtR, waist-to-height ratio; BMI, body mass index; BFP, body fat percentage.

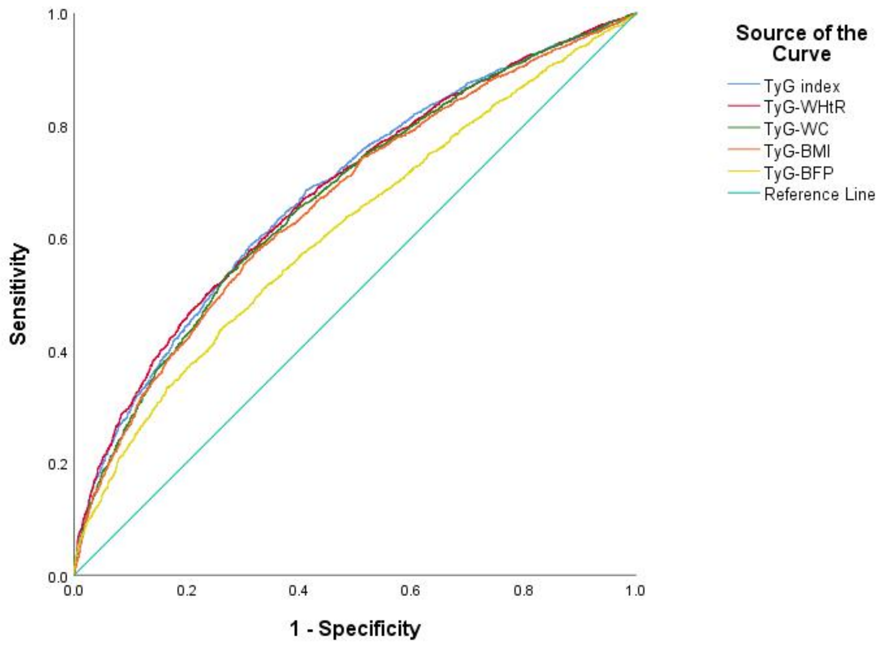

Supplementary Figure 1. Predictive ability of impaired glucose metabolism in TyG and obesity-related TyG indices of different phenotypes.

Abbreviations: TyG index, triglyceride-glucose index; WHtR, waist-to-height ratio; WC, waist circumference; BMI, body mass index; BFP, body fat percentage.
